# Effects of marital status on survival of medullary thyroid cancer stratified by age

**DOI:** 10.1002/cam4.4388

**Published:** 2021-11-01

**Authors:** Lei Ai, Ning Li, Hai‐Long Tan, Bo Wei, Ya‐Xin Zhao, Pei Chen, Hui‐Yu Hu, Mian Liu, Deng‐Jie Ou‐Yang, Zi‐en Qin, Peng Huang, Shi Chang

**Affiliations:** ^1^ Department of General Surgery Xiangya Hospital Central South University Changsha Hunan P.R. China; ^2^ National Clinical Research Center for Geriatric Disorders Xiangya Hospital Central South University Changsha Hunan P.R. China; ^3^ Clinical Research Center For Thyroid Disease In Hunan Province Changsha Hunan P.R. China

**Keywords:** cancer prognosis, marital status, MTC, SEER database, social support

## Abstract

**Purpose:**

Marital status has emerged as an important influence on several cancer outcomes, but its role in medullary thyroid cancer (MTC) remains unclear. This study was to explore the effects of marital status on the prognosis of MTC patients and to determine whether its effects vary by age.

**Patients and methods:**

We retrospectively extracted 1344 eligible patients diagnosed with MTC between 2004 and 2015 from the Surveillance, Epidemiology, and End Results (SEER) database. Based on the marital status, we divided those patients into married and unmarried groups. We compared the difference in overall survival (OS) and cancer‐specific survival (CSS) between married and unmarried via the Kaplan–Meier analysis. Univariate and multivariate Cox proportional models were performed to identify the prognostic factors of OS and CSS.

**Results:**

There were 1344 MTC eligible patients in a total of which 883 (65.7%) were married and 461 (34.3%) were unmarried. The comparison observed between married and unmarried patients was as follows: male (45.2% vs. 28.0%), age (≥52 years) (55.9% vs. 44.6%), White (86.7% vs. 78.7%), and undergo surgery (97.7% vs. 93.3%). Multivariate analysis revealed unmarried status as a risk factor independently associated with worse OS (HR: 2.15, 95% CI: 1.59–2.92) rate and CSS (HR: 1.70, 95% CI: 1.17–2.47) rate. In a further analysis stratified by age, there was no significant difference in OS and CSS between married and unmarried patients younger than 52 years. For the remaining group with 52 years old and higher, unmarried patients showed significantly higher risk of OS and CSS than married patients at all stages of the pathology except M1 stage.

**Conclusion:**

Married patients with MTC have a better prognosis than unmarried ones. Age can affect the association between marital status and the survival of MTC, and married elders may benefit more than youngers.

## Graphical abstract

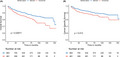

## 1 INTRODUCTION

Medullary thyroid cancer (MTC) is a rare malignancy, which originates from the parafollicular C cells of the thyroid gland, accounting for nearly 3%–5% of all thyroid cancers.^1^, ^2^ Most MTC cases are sporadic, and 20%–30% of cases are multiple endocrine neoplasia type 2 (including MEN2A and MEN2B) or familial.^3^, ^4^ Despite low incidence, the 10‐year overall survival rate of regional MTC was estimated at approximately 75% and decreased to 40% in patients with metastasis.^5^ Early diagnosis and surgery are effective methods to improve both cure and survival rates. Moreover, novel approaches include targeted agents that are proven means of antitumor therapeutics's for clarity. Vandetanib and cabozantinib are used as the evidence‐based treatment of advanced MTC.^6^, ^7^ However, a common limitation of targeted drug therapy is to develop drug resistance and this phenomenon is independent of the type of tumor.^8^

Previous studies have intensively predicted the prognosis of MTC, mainly limiting to the clinicopathological characteristics and therapeutic strategies.^9^, ^10^, ^11^ However, with the growing understanding of human health and disease, more attention is being paid to socio‐psychological factors. A previous clinical trial found that psychosocial factors were linked with low back pain and may affect the prognosis.^12^ Marital status is recognized as one type of socio‐psychological factor that influences the cancer survival.^13^ Extensive studies focused on marital status and cancers showed that married patients have significantly better survival compared to unmarried ones.^14^, ^15^, ^16^ Nonetheless, in analyses to present, few studies on marital status among MTC patients have been conducted.

The Surveillance, Epidemiology, and End Results (SEER) program collects the data of 18 registries on cancer diagnosis, treatment, and survival for nearly 30% of the US population.^17^ It creates a shared research field, thus we can easily analyze the effect of marital status among different cancers. In this study, we analyzed the data from MTC patients using the SEER database. Our work aims to explore the effects of marital status in MTC, especially for older patients.

## 2 MATERIALS AND METHODS

### 2.1 Study population

The data of MTC patients were downloaded from the SEER*Stat Database, version 8.3.6. Patients were chosen for this study if they met the following criteria: (1) Primary sites defined by the International Classification of Diseases for Oncology (ICD‐O‐3), code C73.9. (2) Patients diagnosed with primary cancer from 2004 to 2015. (3) Histological codes were limited to MTC (8345, 8510). Exclusion criteria included: (1) Patients aged less than 18. (2) Patients with incomplete clinical characteristics and treatment. (3) Patients with incomplete demographic and follow‐up information. Finally, 1344 eligible patients were selected for analysis according to inclusion and exclusion criteria.

### 2.2 Study variables

Study variables included sex, age at diagnosis, race, marital status, tumor stage, nodal stage, metastasis, surgery, survival months, and vital status. Age was considered as a continuous variable, measured by means and standard deviations. X‐tile software (version 3.6.1) was used to analyze the best cut‐off point (52 year old) for the age.^18^ After that, two age groups were defined as more than 52 years versus those aged 52 years and lower. The race was divided into three groups: White, Black, and Others (Asian or Pacific Islander, American Indian/Alaska Native). Marital status was categorized into married and unmarried (divorced, separated, widowed, never married, or domestic partner). Thyroid surgery was classed into three groups: none, lobectomy/isthmectomy, and total thyroidectomy. For tumor (T), nodal (N), and metastasis (M) status, the TNM status was assessed according to the sixth edition of TNM classification for medullary thyroid cancer. Overall survival (OS) and cancer‐specific survival (CSS) were also analyzed for all eligible patients. The former was calculated from the date of diagnosis to the date of any death, while the latter was estimated from diagnosis to cancer‐specific caused death.

### 2.3 Statistical analysis

Baseline data were expressed as frequency or mean ± standard deviation according to the data type. Categorical variables were assessed by the Pearson chi‐squared test, while the continuous variables were examined by the *t*‐test or the Mann–Whitney *U* test. The survival of marital status and age subgroups was analyzed by the Kaplan–Meier curves, and their differences were evaluated by the log‐rank test. Univariate and multivariate Cox proportional hazards models were used to distinguish the independent prognostic factors in MTC, and their effects were presented as hazard ratio (HR) with 95% confidence intervals (CIs). Similarly, we evaluated the effects of marital status stratified by age, using the multivariate Cox proportional hazards models to analyze the survival difference between married and unmarried in pathological subgroups. All statistical analyses in this study were performed using SPSS (version 26.0) and R software (version 4.1.0). A two‐tailed p value of less than 0.05 was considered statistically significant.

## 3 RESULTS

### 3.1 Patients baseline characteristics

We selected 1344 eligible MTC patients diagnosed between 2004 and 2015 in the SEER database. It included 532 (39.3%) male and 816 (60.7%) female patients, with a mean age of 52.9 ± 15.5 years at the diagnosis of MTC. Among them, 883 (65.8%) were married and 461 (34.2%) were unmarried. Between married and unmarried groups, we observed significant differences in sex, age, race, and surgery (all *p* < 0.001). Besides, married patients were male (45.2% vs. 28.0%), older (age ≥ 52) (55.9% vs. 44.6%), White (86.7% vs. 78.7%), more likely to undergo surgery (97.7% vs. 93.3%), and less to be in M1 status (7.2% vs. 10.2%) compared to unmarried patients. The summary of baseline patient characteristics grouped by marital status is described in detail in Table 1.

**TABLE 1:** Baseline characteristics of patients with MTC in SEER database

Characteristics	Total (%)	Married (%)	Unmarried (%)	*p* value^b^
Number	*N* = 1344 (100)	*N* = 883 (65.7)	*N* = 461 (34.3)	
Sex				
Female	816 (60.7)	484 (54.8)	332 (72.0)	**<0.001**
Male	528 (39.3)	399 (45.2)	129 (28.0)	
Age^a^				
Mean ± SD	52.9 ± 15.5	54.0 ± 14.1	50.7 ± 17.9	**<0.001**
<52	637 (47.4)	389 (44.1)	248 (53.4)	
≥52	707 (52.6)	494 (55.9)	213 (46.6)	
Race				
White	1129 (84.0)	766 (86.7)	363 (78.7)	**<0.001**
Black	127 (9.4)	53 (6.0)	74 (16.1)	
Others	88 (6.5)	64 (7.3)	24 (5.2)	
Tumor stage				
T1	581 (43.2)	365 (41.3)	216 (46.9)	0.281
T2	347 (25.8)	234 (26.5)	113 (24.5)	
T3	282 (21.0)	193 (21.9)	89 (19.3)	
T4	134 (10.0)	91 (10.3)	43 (9.3)	
Nodal stage				
No	783 (58.3)	513 (58.1)	270 (58.6)	0.877
N1a	166 (12.4)	107 (12.1)	59 (12.8)	
N1b	395 (29.4)	263 (29.8)	132 (28.6)	
Metastasis				
M0	1233 (91.7)	819 (92.8)	414 (89.8)	0.062
M1	111 (8.3)	64 (7.2)	47 (10.2)	
Surgery				
No	51 (3.8)	20 (2.3)	31 (6.7)	**<0.001**
IT/LT	94 (7.0)	63 (7.1)	31 (6.7)	
TT	1199 (89.2)	800 (90.6)	399 (86.6)	

Variables with statistical significance were shown in bold. Abbreviations: IT/LT, isthmectomy/lobectomy; MTC, medullary thyroid cancer; SEER, The Surveillance, Epidemiology, and End Result; TT: total thyroidectomy or near total thyroidectomy.

^a^ Age was a continuous variable and grouped by cut‐off point using x‐tile software.

^b^ Categorical variables were assessed by the chi‐squared test and continuous variables were examined by the Mann–Whitney *U* test.

### 3.2 Effects of marital status on OS and CSS

**FIGURE 1:**
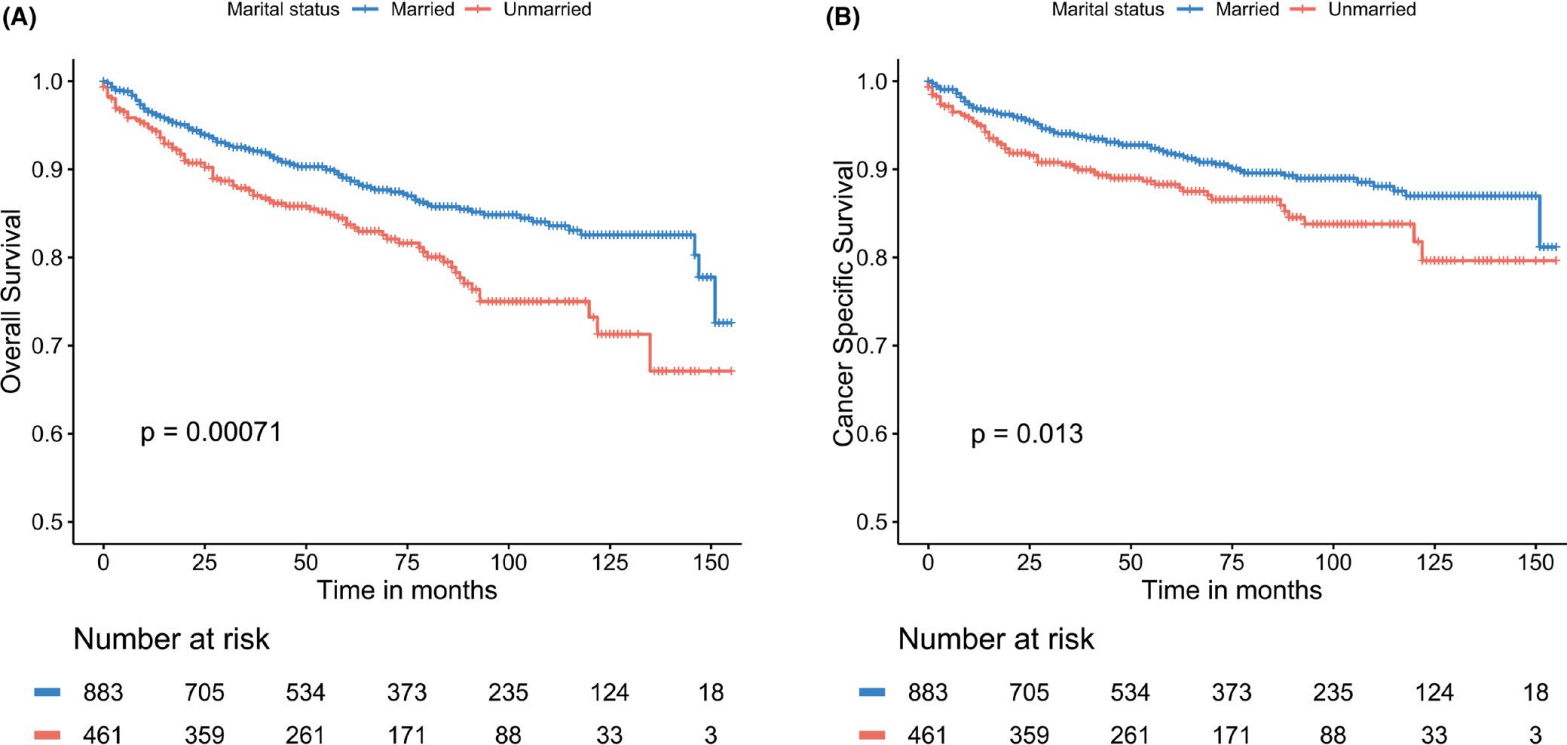
The Kaplan–Meier survival curves: (A) The overall survival and (B) the cancer‐specific survival according to marital status

The effects of marital status on OS were examined by K‐M curves, which showed married patients had a significantly superior OS compared to unmarried ones (Figure 1A). Similarly, the significant differences of CSS were also observed among different marital groups (Figure 1B) with a better CSS in married populations than in unmarried ones. To evaluate the prognosis‐related factors of MTC, we first performed the univariate cox analysis. The results showed that marital status, sex, age, tumor stage, nodal stage, metastasis, and surgery were regarded as significant prognostic factors for both OS (all *p* < 0.05) (Table 2) and CSS (all *p* < 0.05) (Table 3). Race was a prognostic factor for CSS (*p* < 0.05), but not OS (*p* > 0.05) in univariate analysis. Subsequently, those above‐mentioned significant factors were analyzed in a multivariate cox model. After multivariate adjustment, marital status remained a significant prognostic factor in OS (*p* < 0.001) and CSS (*p* = 0.006), with worse OS (HR: 2.15, 95% CI: 1.59–2.92) rate and CSS (HR: 1.70, 95% CI: 1.17–2.47) rate in unmarried patients compared to married ones. However, there was no significant survival difference observed in sex (OS: *p* = 0.858, CSS: *p* = 0.826).

**TABLE 2:** Univariate and multivariate analyses for OS in MTC patients

Variables	Univariate analysis		Multivariate analysis	
	HR (95% CI)	*p* value	HR (95% CI)	*p* value
Sex				
Female	Reference		Reference	
Male	1.61 (1.21–2.14)	**0.001**	0.97 (0.71–1.33)	0.858
Age^a^				
<52	Reference		Reference	
≥52	3.58 (2.55–5.02)	**<0.001**	4.33 (3.06–6.13)	**<0.001**
Race				
White	Reference			
Black	1.22 (0.78–1.91)	0.373		
Others	0.50 (0.22–1.12)	0.093		
Marital status				
Married	Reference		Reference	
Unmarried	1.63 (1.23–2.17)	**0.001**	2.15 (1.59–2.92)	**<0.001**
Tumor stage				
T1	Reference		Reference	
T2	1.86 (1.18–2.93)	**0.007**	1.86 (1.18–2.94)	**0.008**
T3	3.54 (2.33–5.38)	**<0.001**	2.26 (1.42–3.59)	**0.001**
T4	9.65 (6.39–14.58)	**<0.001**	3.96 (2.43–6.44)	**<0.001**
Nodal stage				
N0	Reference		Reference	
N1a	2.26 (1.44–3.54)	**<0.001**	1.77 (1.10–2.86)	**0.019**
N1b	4.11 (2.99–5.65)	**<0.001**	1.84 (1.22–2.76)	**0.004**
Metastasis				
M0	Reference		Reference	
M1	11.50 (8.51–15.54)	**<0.001**	3.86 (2.65–5.61)	**<0.001**
Surgery				
No	Reference		Reference	
IT/LT	0.06 (0.03–0.13)	**<0.001**	0.31 (0.14–0.67)	**0.003**
TT	0.08 (0.06–0.12)	**<0.001**	0.30 (0.20–0.46)	**<0.001**

Variables with statistical significance were shown in bold. Abbreviations: IT/LT, isthmectomy/lobectomy; MTC, medullary thyroid cancer; OS, overall survival; TT: total thyroidectomy or near total thyroidectomy.

^a^ Age was a continuous variable and grouped by cut‐off point using x‐tile software.

**TABLE 3:** Univariate and multivariate analyses for CSS in MTC patients

Variables	Univariate analysis		Multivariate analysis	
	HR (95% CI)	*p* value	HR (95% CI)	*p* value
Sex				
Female	Reference		Reference	
Male	2.05 (1.46–2.88)	**<0.001**	0.96 (0.66–1.39)	0.826
Age^a^				
<52	Reference		Reference	
≥52	2.48 (1.71–3.59)	**<0.001**	2.96 (2.01–4.38)	**<0.001**
Race				
White	Reference		Reference	
Black	1.32 (0.79–2.19)	0.291	1.90 (1.11–3.23)	**0.019**
Others	0.11 (0.02–0.81)	**0.030**	0.10 (0.01–0.70)	**0.021**
Marital status				
Married	Reference		Reference	
Unmarried	1.53 (1.09–2.16)	**0.014**	1.70 (1.17–2.47)	**0.006**
Tumor stage				
T1	Reference		Reference	
T2	2.36 (1.24–4.49)	**0.009**	2.23 (1.16–4.28)	**0.016**
T3	6.03 (3.40–10.72)	**<0.001**	2.86 (1.53–5.33)	**0.001**
T4	19.17 (10.98–33.48)	**<0.001**	5.24 (2.78–9.86)	**<0.001**
Nodal stage				
N0	Reference		Reference	
N1a	3.93 (2.21–6.99)	**<0.001**	2.81 (1.52–5.20)	**0.001**
N1b	7.98 (5.15–12.36)	**<0.001**	2.77 (1.62–4.73)	**<0.001**
Metastasis				
M0	Reference		Reference	
M1	19.56 (13.89–27.54)	**<0.001**	6.05 (3.95–9.25)	**<0.001**
Surgery				
No	Reference		Reference	
IT/LT	0.04 (0.02–0.10)	**<0.001**	0.34 (0.13–0.93)	**0.036**
TT	0.06 (0.04–0.10)	**<0.001**	0.32 (0.20–0.50)	**<0.001**

Variables with statistical significance were shown in bold. Abbreviations: CSS, cancer‐specific survival; IT/LT, isthmectomy/lobectomy; MTC, medullary thyroid cancer; TT: total thyroidectomy or near total thyroidectomy.

^a^ Age was a continuous variable and grouped by cut‐off point using x‐tile software.

### 3.3 Effects of marital status on OS and CSS according to age stratification

**FIGURE 2:**
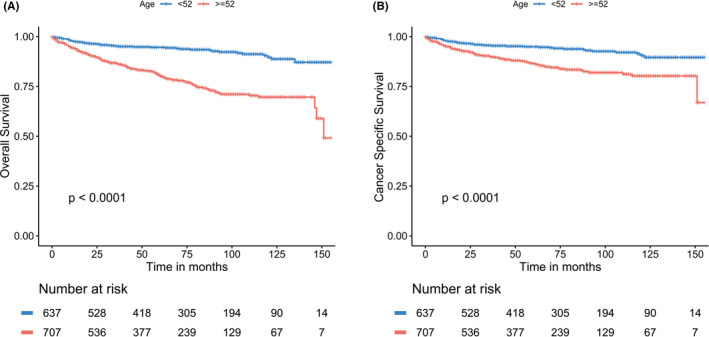
The Kaplan–Meier survival curves: (A) The overall survival and (B) the cancer‐specific survival according to age

We first assessed the association between age and survival by the Kaplan–Meier curves, which indicated that older patients were more likely to present worse survival compared to youngers (OS: *p* < 0.001; CSS: *p* < 0.001) (Figure 2A,B). Furthermore, to determine the effects of marital status on the survival of different pathological stages varies with age. We compared the OS and CSS of unmarried versus married based on age stratification by multivariate cox models, which were adjusted for the sex, race, marital status, TNM stage, and surgery. As shown in Table 4, marital status had no effect on survival in MTC patients younger than 52 years of age. In older patients, however, marital status had a significant effect on survival at all pathological subgroups except M1 stage, with unmarried groups presented a higher risk of OS and CSS (all *p* < 0.05) compared to married ones. These results showed that marriage had a significant protective effect in older patients among different pathological stages and its effect declined when the tumor progressed.

**TABLE 4:** The OS and CSS associated with being unmarried (vs. married) among MTC patients stratified by age, sex, and pathological stages

Age^a^	OS		CSS	
	HR (95% CI)^b^	*p* value	HR (95% CI)^b^	*p* value
<52				
Sex				
Female	0.90 (0.35–2.31)	0.834	1.07 (0.42–2.68)	0.892
Male	1.29 (0.19–3.39)	0.613	1.29 (0.19–3.39)	0.613
Tumor stage				
T1/T2	1.86 (0.55–6.28)	0.317	3.27 (1.03–10.42)	**0.045**
T3/T4	1.03 (0.49–2.18)	0.932	0.95 (0.44–2.06)	0.895
Nodal stage				
N0	0.78 (0.08–7.17)	0.829	1.52 (0.15–15.38)	0.720
N1	1.13 (0.55–2.33)	0.732	1.14 (0.54–2.41)	0.740
Metastasis				
M0	0.94 (0.41–2.16)	0.883	1.03 (0.42–2.54)	0.944
M1	2.67 (0.60–11.78)	0.196	2.67 (0.60–11.78)	0.196
≥52				
Sex				
Female	1.96 (1.21–3.18)	**0.007**	1.17 (0.60–2.28)	0.649
Male	2.99 (1.75–5.10)	**<0.001**	3.39 (1.83–6.30)	**<0.001**
Tumor stage				
T1/T2	2.80 (1.62–4.85)	**<0.001**	2.90 (1.33–6.34)	**0.007**
T3/T4	2.32 (1.40–3.82)	**0.001**	2.16 (1.23–3.79)	**0.007**
Nodal stage				
N0	3.32 (1.84–5.99)	**<0.001**	3.36 (1.38–8.21)	**0.008**
N1	1.76 (1.10–2.81)	**0.018**	1.62 (0.95–2.77)	0.074
Metastasis				
M0	2.44 (1.57–3.78)	**<0.001**	1.97 (1.01–3.84)	**0.046**
M1	1.74 (0.90–3.38)	0.101	1.78 (0.92–3.49)	0.087

Variables with statistical significance were shown in bold. Abbreviations: CSS, cancer‐specific survival; MTC, medullary thyroid cancer; OS, overall survival.

^a^ Age was a continuous variable and grouped by cut‐off point using x‐tile software.

^b^ Models were adjusted for sex, marital status, race, tumor stage (T1/T2, T3/T4), nodal stage (N0, N1), metastasis (M0, M1), and surgery.

## 4 DISCUSSION

In this study, we investigated the association between marital status and survival based on a large cohort of MTC patients. Since age is an important factor affecting thyroid cancer outcomes, we also evaluated the effects of marital status at different age groups on MTC outcomes.^19^ To our knowledge, this is the first study to explore the interaction of age and marital status in MTC survival.

In our study, we found that married patients showed a lower M1 stage of tumor compared to unmarried ones, but opposite results were observed in the T stage. Moreover, only 20 (2.3%) married patients were not treated with surgery, which is significantly lower than 31 (6.7%) of unmarried patients. In univariate analysis, we identified marital status as an independent prognostic factor, with unmarried patients showed a higher risk of death. After adjustment for demographic and pathological characteristics, increased death risk was also found in unmarried patients compared to married ones. In addition, we investigated the effects of marital status in different pathological stages and sex groups based on age. Our results demonstrated that marriage has a significant protective effect on older (age ≥ 52) patients, while this effect decreased with the progression of the tumor. However, we found no significant difference in OS and CSS between married and unmarried patients younger than 52 years. In the previous studies, married younger patients were found to have significant better outcomes than unmarried ones in some other cancers, including breast cancer, multiple myeloma, and oral cavity cancer.^20^, ^21^, ^22^, ^23^ But, a latest study focused on differentiated thyroid cancer (DTC) showed the impact of marital status on survival varied with age, which was similar to our results.^24^ It is well known that the prognosis of thyroid cancer is usually better in younger patients than older ones, thus we suggest that there is an interaction between age and marital status. In younger patients, the benefits of age may far beyond the benefits of marital status, which masked the survival difference between married and unmarried, and the opposite was in old patients.

The association of marital status and survival was explored in many tumors, including breast cancer, rectal cancer, and non‐small cell lung cancer.^14^, ^15^, ^16^ Consistent with the findings of the above researches, our results presented that marriage was a factor associated with superior survival. In a previous study focusing on differentiated thyroid cancer patients, Shi et al. found unmarried patients increased the risk of tumor mortality.^24^ A study of breast cancer showed greater protection of marriage among patients aged 70 years or older.^20^ By collating and analyzing more than a million patients diagnosed with different cancers, unmarried patients presented a higher risk of metastatic cancer and death resulting from cancer.^13^

Two possibilities could explain the superior survival of married patients than unmarried ones. On the one hand, these married patients were supervised by their spouses for regular physical examinations before being diagnosed, which contributes to the early detection of MTC. Spouses may also provide more economic support for subsequent treatments. In MTC, early stage surgical intervention has a benefit for survival, because the surgical cure is possible for patients without metastasis or with regional lymph nodes confined to the neck.^25^ However, the therapeutic efficacy of surgery is limited when the tumor metastasizes outside the neck. Thus, married patients could obtain a better prognosis according to the early surgery.

On the other hand, the psychological disorder is common among cancer patients with more than four times higher than the ordinary individuals.^26^ Recent studies showed that patients obtaining a cancer diagnosis have a greater susceptibility to develop psychiatric disorders, such as stress, depression, anxiety, etc.^27^, ^28^ However, married people showed less depression and psychological distress after being diagnosed with cancer, which may be attributed to the encouragement and support from their spouses.^29^ The impact of psychological distress on tumor progression has been implicated by epidemiological studies.^30^ For example, stress exposure promotes tumor cell proliferation, angiogenesis, and invasion by stimulating the adrenergic pathway.^31^ Additionally, the cortisol circadian rhythm of patients is changed due to cancer‐related psychological stress, which will cause poor survival.^32^ Some cytokines that are involved in inflammation response, including IL‐1β, IL‐6, TNF, and C‐reactive protein (CRP), are increased in depressed patients and promote tumor progression.^33^, ^34^ Therefore, the lack of social support and suffering from psychological problems perhaps partly explain the higher mortality in unmarried patients.

Despite these findings between marital status and survival, there remain several limitations in our study. First, an inherent limitation is related to our retrospective study, limiting our ability to define a cause‐and‐effect relationship between marital status and survival. Second, marital status in the SEER database was recorded at diagnosis. However, marital status is dynamic and may have changed throughout the follow‐up period, thus affecting the final results. In addition, the quality of marriage was not recorded, which may lead to different survival outcomes. Third, some information regarding socioeconomic status and education is not available in the SEER database. Finally, data on the type of chemotherapy or targeted agents are unavailable and thus are not included in our study, which may bias our present results.

## 5 CONCLUSION

In summary, our present results showed marital status was an independent prognostic factor in MTC. Unmarried patients showed a higher risk of OS and CSS, which is particularly evident in older patients. Further studies could investigate the mechanism for age affecting the benefits of marriage. Returning to our research, unmarried patients (especially the old unmarried patients) need to be noticed with more social care and psychological support to improve their survival.

## CONFLICT OF INTEREST

The authors declare no conflict of interest in this work.

## ETHICAL APPROVAL STATEMENT

No ethical approval is required in this study.

## Data Availability

The dataset of this study is available in the Surveillance, Epidemiology, and End Results program (www.seer.cancer.gov) SEER*Stat Database: Incidence–SEER 18 Regs Custom Data (with additional treatment fields), Nov 2018 Sub (1975–2016 varying).
